# 

**Published:** 1871-01

**Authors:** 


### INDEX OF SUBJECTS.

### ORIGINAL ARTICLES AND SPECIAL SELECTIONS:

Fulton County Medical Society........................................................ 1
Chicago Medical Society........................................................ 2
Cincinnati Academy of Medicine........................................................ 8
Nashville Correspondence........................................................ 10
Glycerine of Tannin........................................................ 12
Permanganate of Potassa in the Treatment of Certain Female Diseases........................................................ 13
Use of Arsenic in Phthisis........................................................ 14
Management of the Placenta........................................................ 15
Chloride of Soda for Burns, Ulcers, etc.—Alum as a Remedy in Croup—Assafœtida in Whooping Cough—Treatment for Ulceration of Stomach and Cancreum Oris........................................................ 16

### EXTRACTS AND GLEANINGS.

Treatment of Asthma, 17; Treatment of Pulmonary Consumption, 18; Scarlet Fever—Strychnine in Intermittent Fevers—20; Bromide of Ammonium in Menorrhagia—Nitrate of Silver in Orchitis—Chloroform as a Febrifuge and Anti-Periodic—Remedies for Gonorrhœa—21; Remedy for Chronic Inflammation of Bladder—Perchloride of Iron in Post-Partum Hemorrhage—Bromide of Potassium in Puerperal Convulsions—Hypophosphites in Toothache of Pregnancy—22; Carbolic Acid in Sickness of Pregnancy—Ergot in Retention of Urine—Liquor Potassæ in Stranguary—Carbolic Acid in Snake Bites—Antidote for Carbolic Acid Poisoning—Digitalis in Consumption—Hydrate of Choral in Cerebro-Spinal Meningitis—23; Chestnut Leaves in Whooping Cough—Sulphur in Diphtheritic Angina—Tannic Acid in Fissures of Anus—Creosote in Mercurial Salivation—Remedy for Sleeplessness—Remedy for Ring Worm—Death from Chloroform—24; Prescription for Epilepsy—Nitrate of Lead in Sore Nipples—Loss of Speech after Chloroform—25; Prescription for Secondary and Tertiary Syphilis—Carbolic Acid in Gleet—Bromide of Lithium in Epilepsy—26.

### EDITORIALS. REVIEWS, ITEMS, AND NEWS.

Our Journal, 27; The American Medical Association, 30; The Massachusetts Medical Society, 32; Letter of a Valued Correspondent, 34; Our First Number, 36; Our Advertising Friends—A new Feature of our Journal, 37; University of Louisville, 38; Our Exchanges, 40; An Apology—Medical Society of the District of Columbia, 42.

